# Use of mobile phones for behavior change communication to improve maternal, newborn and child health: a scoping review

**DOI:** 10.7189/jogh.09.020425

**Published:** 2019-12

**Authors:** Alison Mildon, Daniel Sellen

**Affiliations:** 1 Department of Nutritional Sciences, University of Toronto, Toronto, Ontario, Canada; 2 Joannah & Brian Lawson Centre for Child Nutrition, University of Toronto, Toronto, Ontario, Canada

## Abstract

**Background:**

Behavior change communication (BCC) to improve health and caring practices is an integral component of efforts to improve maternal, newborn and child health (MNCH). Mobile phones are widely available in low- and middle-income countries (LMIC), presenting new opportunities for BCC delivery. There is need for delivery science to determine how best to leverage mobile phone technology for BCC to improve MNCH practices.

**Methods:**

We conducted a scoping review of studies and project reports documenting the feasibility, implementation or effectiveness of using mobile phones for BCC delivery related to MNCH in LMIC. Data were extracted and synthesized from three sources: i) systematic search of three electronic databases (PubMed, MedLine, Scopus); ii) grey literature search, including mHealth databases and websites of organizations implementing mHealth projects; iii) consultation with researchers and programme implementers. Records were screened using pre-determined inclusion criteria and those selected were categorized according to their primary intervention delivery approaches. We then performed a descriptive analysis of the evidence related to both effectiveness and implementation for each delivery approach.

**Results:**

The systematic literature search identified 1374 unique records, 64 of which met inclusion criteria. The grey literature search added 32 records for a total of 96 papers in the scoping review. Content analysis of the search results identified four BCC delivery approaches: direct messaging, voice counseling, job aid applications and interactive media. Evidence for the effectiveness of these approaches is growing but remains limited for many MNCH outcomes. The four approaches differ in key implementation elements, including frequency, length and complexity of communication, and potential for personalization. These elements influence resource allocation and are likely to impact effectiveness for BCC targeting complex, habitual MNCH practices.

**Conclusions:**

This scoping review contributes to the evidence-base on the opportunities and limitations of using mobile phones for BCC delivery aiming to improve MNCH practices. The incorporation of mobile phone technology in BCC interventions should be guided by formative research to match both the content and delivery approach to the local context. We recommend five areas for further research, including both effectiveness and implementation studies on specific delivery approaches.

Improved caring practices is one important pathway towards progress in many aspects of maternal, newborn and child health (MNCH) in low- and middle-income countries (LMIC). Behavior change communication (BCC) has become an integral component of many MNCH interventions, but there are multiple known challenges to its implementation [1], particularly when addressing complex, habitual practices that are influenced by a variety of contextual factors [2].

The proliferation of mobile phones in LMIC suggests great potential to dramatically expand information access and enhance engagement with health messaging using mobile phone technology for health (mHealth) [3,4]. The current state of knowledge is fragmented and challenging to synthesize because there are numerous deployments of new mHealth initiatives and the timeliness and quality of reporting and assessment varies [5]. There is a need to strengthen the research agenda for evaluating such initiatives, and specifically to categorize BCC delivery approaches to support improved MNCH practices in LMIC. To meet this practical and scientific need, we present key findings of a broad scoping review that aimed to categorize BCC delivery approaches, and to describe the current state of evidence for the major approaches identified.

Globally, there are an estimated 5.7 billion unique individual mobile phone subscribers, with significant continued growth in LMIC [6]. There is optimism that leveraging mobile phone technology will assist in overcoming barriers to health system functioning and service delivery in LMIC, and many projects are integrating mHealth components [4,7,8]. Within the broader field of digital health, the term “mHealth” is used to refer collectively to the use of mobile technology for health-related functions, including data collection and management, service delivery, health communication and diagnostics [5,9]. Mobile phones are the primary mHealth technology and the most relevant to BCC interventions.

To our knowledge, two systematic reviews of mHealth for BCC in LMIC have been published. In 2012, Gurman et al. assessed sixteen studies of varying designs for their adherence to best practice guidelines for mHealth intervention designs [10]. Most showed evidence of appropriate formative work but tailoring of messages was limited, and few studies included long-term follow up or evaluation. More recently, Higgs et al. identified fifteen studies, primarily randomized controlled trials (RCTs), reporting causal attribution of mHealth interventions to behavioral outcomes relevant to child survival [11]. All selected studies used text messaging as the BCC intervention and addressed episodic rather than complex, habitual health behaviors. No conclusions regarding effectiveness could be drawn from either of these reviews. The authors of both reviews called for more attention to building the mHealth evidence base and highlighted specific limitations of the existing data in the field as the small number of available studies and the heterogeneity of study contexts, intervention strategies and target health outcomes. Higgs et al. also highlighted the need for implementation research to identify mechanisms of change and contextual factors influencing the success of mHealth interventions [11]. The mHealth evidence base has grown substantially since these two reviews were published, enabling some of the identified limitations to be addressed through an updated review.

Despite this growth, heterogeneity of mHealth study designs and contexts continue to hinder the determination of effects on MNCH outcomes. A systematic review of mHealth interventions targeted to pregnant women reported improvements in antenatal and neonatal service utilization, but evidence of effects on maternal and neonatal outcomes was limited, preventing meta-analysis [12]. A comprehensive systematic review of the effectiveness of mHealth interventions for MNCH outcomes in LMIC identified only fifteen relevant studies, ten of which were BCC interventions [13]. Meta-analysis was conducted for three studies which aimed to improve exclusive breastfeeding practices [14-16]. There were positive effects of the mHealth interventions on early initiation of breastfeeding (odds ratio OR = 2.01; 95% confidence interval (CI) = 1.27-2.75) and exclusive breastfeeding at three or four months (OR = 1.88; 95% CI = 1.26-2.50) and at six months (OR = 2.57; 95% CI = 1.46-3.68) [13]. The evidence review for the recently published Digital Health Guidelines found moderate evidence that targeted client communication improves attendance at four or more antenatal care (ANC) visits, antenatal use of iron-folic acid supplements, skilled birth attendance and infant vaccination rates, but concluded that effects on other MNCH outcomes are uncertain due to limited or poor quality evidence [5].

Notwithstanding the limited evidence base, multiple projects utilizing mHealth for MNCH are being designed and implemented within the programming sphere. These initiatives provide a rich basis for implementation learning that is not captured in systematic reviews of the peer reviewed scientific articles focused on determining effectiveness. In addition, mHealth interventions vary considerably in their approaches. There is a need to examine the characteristics, strengths and limitations of specific mHealth delivery approaches, as an aggregate effect size may be less useful for identifying specific strategies that could be implemented to address particular MNCH practices. The aims of this research were therefore: i) to classify, based on current evidence from recent and ongoing projects and programs, the specific approaches through which mobile phones can be used to deliver BCC related to MNCH; and ii) to describe the current state of evidence related to both effectiveness and implementation for each delivery approach. Within the broad field of MNCH this work focuses on BCC interventions intended to improve home-based care practices and uptake of preventive health services for mothers and young children during pregnancy, infancy and the first five years of childhood. It complements a separate forthcoming analysis that adopts a particular focus on infant and young child feeding.

## METHODS

We followed Arksey and O’Malley’s five-stage methodology for scoping reviews in the health sciences [17]. Data were collected through searches of both the published and grey literature, and consultation with researchers and implementers of mHealth projects targeting improved MNCH practices. Ethics approval was not required.

Three major online databases (PubMed, MedLine and Scopus) were searched using the key term “mobile phone” with the following search terms: “counseling”, “behavior change”, “nutrition”, “infant feeding”, and “breastfeeding”. The full PubMed search is provided in Appendix S1 in **Online Supplementary Document**, and formed the basis of the MedLine and Scopus searches. These terms were chosen to achieve both a broad review of the relevant BCC literature and to address our specific interest in infant and young child feeding. Inclusion of the terms “maternal”, “neonatal”, “infant”, and “child” with the core search terms was tested in PubMed but did not produce any additional results, so these terms were not incorporated into the full search strategy. The search was initially conducted in October 2015 and subsequently updated to include papers published up to December 31, 2018. Searches were filtered for English language papers only, and the Scopus search was limited to journals in the medical and social science fields. All records were exported to EndNote X7 (Thomson Reuters, Toronto ON, Canada) citation management software and duplicates were removed.

To identify any additional BCC interventions within the target definition that may not have been reported in the peer-reviewed, published and indexed scientific literature we searched for relevant grey literature in online repositories of mHealth program information using the terms “behavior change”, “nutrition” and “counseling” separately and in combination. We also searched the websites of many organizations known to be engaged in mHealth, either using the website’s internal search function or by screening the documents available for download under relevant website tabs. These online repositories and websites are listed in Tables S1 and S2 in **Online Supplementary Document**. Additional published studies and grey literature reports were identified through review of the reference lists of all selected documents, PubMed suggestions for similar articles, Google searches, and through direct consultation with implementers and researchers in the mHealth field. We also searched two registries of clinical trials (https://clinicaltrials.gov and the WHO International Clinical Trials Registry, http://apps.who.int/trialsearch/Default.aspx) using the terms “mHealth”, “mobile phone” and “mobile health” combined with “nutrition”, “behavior change” and “counseling” to identify current research studies relevant to the scoping review. New papers were added to the grey literature search until February 8, 2019.

We screened all records from these literature searches using pre-determined inclusion criteria. Published articles were selected if they reported on feasibility or intervention studies in LMIC related to the use of mobile phone technology for BCC to improve home-based care practices and uptake of preventive health services for mothers and young children during pregnancy, infancy and the first five years of childhood. There were no restrictions by publication date or study methodology. We excluded studies reporting on: monitoring and evaluation aids; electronic health records; clinical interventions; MNCH interventions occurring outside our target period of pregnancy to age five; and interventions in high-income countries. Abstracts were reviewed for records that could not be excluded by title alone; where uncertainty remained, the full text was reviewed.

We reviewed all potentially relevant documents from the grey literature by title, abstract or executive summary (if available), or full text. We included documents that reported on the feasibility, evaluation or implementation lessons from interventions conducted in LMIC using mobile phone technology for BCC to improve home-based care practices and uptake of preventive health services for mothers and young children during pregnancy, infancy and the first five years of childhood. Media articles and blog posts were excluded, as were project briefs without any evaluation data or reflection on lessons learned. Only one grey literature document was included for each discrete mHealth project unless additional documents contributed unique findings.

We categorized the selected studies and reports according to their primary intervention delivery approaches. Data were extracted and compiled using charting tables developed to record key details regarding study methodology (design, sample size, location), intervention, and findings or lessons learned. We performed a descriptive analysis of the evidence related to both effectiveness and implementation for each delivery approach.

## RESULTS

**Figure 1:**
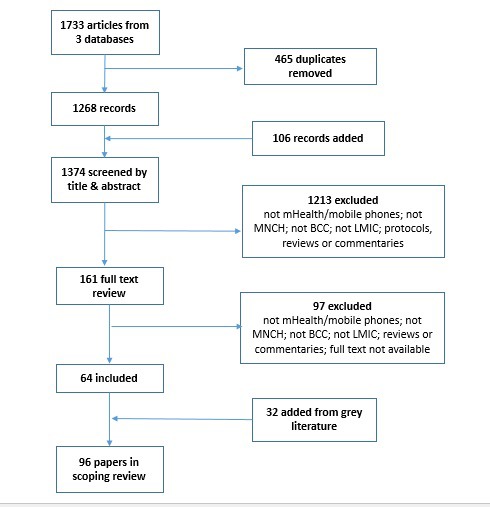
Literature search flowchart.

The search of published literature yielded 1374 unique articles after removal of duplicates. After title and abstract review, 161 remained for full text review, of which 64 met the inclusion criteria. A further 32 were added from the grey literature, including unpublished research and programmatic case studies, for a total of 96 papers included in the scoping review (**Figure 1**).

We classified the papers into four BCC delivery approaches: **direct messaging, voice counseling, job aid applications and interactive media** (**Table 1**). Several projects utilized both direct messaging and voice counseling, and are presented under this combined category. **Table 2, 3, 4, 5** and** 6** provide an overview of the studies and reports included under each delivery approach.

**Table 1:** Literature search results by behaviour change communication delivery channel

Type	Source	Direct messaging	Voice counseling	Messaging + voice	Job aid apps	Interactive media
**Intervention studies**	Published	16	1	3	7	1
	Grey	4	1	3	2	
**Implementation Studies**	Published	9		2	3	
	Grey	1		1		
**Feasibility Studies**	Published	6		2	3	
	Grey					
**Formative/Qualitative Studies**	Published	6	2	1	2	
	Grey			1	1	
**Case studies & other programmatic reports**	Published					
	Grey	8		1	9	
**TOTAL documents**		**50**	**4**	**14**	**27**	**1**

**Table 2:** Direct messaging studies and programmatic reports

Study		Design		Messaging intervention		Target outcomes		Key results
**Intervention studies – published literature:**								
Jiang et al, 2014 [14]		Quasi-experimental cluster randomized trial in Shanghai, China; N = 582 mothers in first trimester recruited from 4 community health clinics		Weekly SMS on infant feeding from 3^rd^ trimester to 12 months postpartum; participants could text questions to a health professional		Increased exclusive breastfeeding (EBF) duration		Median duration EBF: 11.41 weeks (I.) vs 8.87 (C.). EBF at 6 months: 15.1% vs 6.3%
Unger et al, 2018 [18]		Randomized controlled trial (RCT) in Nairobi, Kenya; N = 298 pregnant mothers with own mobile phones.		Weekly SMS from pregnancy to 12 weeks postpartum. Group 2 also had bi-directional SMS communication with a nurse.		Increase facility-based delivery (FBD), EBF duration and postpartum contraception use.		No significant effects for FBD & contraceptive use. EBF probability significantly higher vs Control in both I-groups at 10 weeks (OR = 0.93; 95% CI = 0.86-0.97 and OR = 0.96; 95% CI = 0.89-0.98 vs OR = 0.79; 95% CI = 0.69-0.86; *P* < 0.005) and 16 weeks (OR = 0.82; 95% CI = 0.72-0.89 and OR = 0.93; 95% CI = 0.85-0.97 vs OR = 0.62; 95% CI = 0.52-0.71; *P* < 0.005) and in I-2 at 24 weeks (OR = 0.62; 95% CI = 0.51-0.72 vs OR = 0.41; 95% CI = 0.31-0.51; *P* = 0.005).
Flax et al, 2014 [16]	Cluster RCT in Bauchi state Nigeria; N = 390 mothers pregnant at baseline & interviewed when infants ≥6 months		Intervention × 10 months: monthly large group breastfeeding education; weekly SMS to small group who presented to monthly large group		Increase timely breastfeeding initiation and EBF duration		Timely initiation: OR = 2.6 (95% CI = 1.6-4.1). EBF to 6 months: OR = 2.4 (95% CI = 1.4-4.0)	
Lund et al, 2012 [19]	Wired Mothers project; cluster RCT via 24 health facilities in Zanzibar, Tanzania; N = 2550 (Intervention: n = 1311)		Intervention group received 1-way SMS tips & reminders 2×/months to 36weeks gestation then 2×/week to 6 weeks postpartum; also received airtime voucher & health worker phone number.		Increase in skilled birth attendance		Skilled birth attendance: 60% vs 47% overall but non-significant for rural women. Urban OR = 5.73 (95% CI = 1.51-21.81).	
Lund et al, 2014 [20]	As above		As above		Primary: Increase in mothers attending >4 antenatal care (ANC) visits. Secondary: improve timing and quality of ANC service delivery		4+ANC: 44% I vs 31% C. OR = 2.39 (95% CI = 1.03-5.55). Trend towards improved timing & quality of ANC services but not significant.	
Lund et al, 2014 [21]	As above		As above		Reduced perinatal mortality		Significant difference in perinatal mortality: 19/1000 (I) vs 36/1000 (C). OR = 0.50 (0.27-0.93). Non-significant reduction in stillbirths (OR = 0.65; 95% CI = 0.34-1.24) & deaths in first 42 d of life (OR = 0.79; 95% CI = 0.36-1.74)	
Fedha, 2014 [22]	RCT in 4 ANCs in Kenya. N = 397 pregnant mothers		Fortnightly SMS reminders of ANC visits and pregnancy health information		Increase uptake of ANC services and FBD. Reduce neonatal mortality.		<4 ANC visits: 3.6% (I) vs 9.7% (C) (*P* = 0.002). FBD: 88.0% (I) vs 72.8% (C) (*P* = 0.00). No significant difference in intrauterine deaths (1% vs 1.5%; *P* = 0.715) or neonatal deaths (1.0% vs 3.4%; *P* = 0.269).	
Omole et al, 2016 [23]	Quasi-experimental pre/post study in 4 ANCs in Nigeria; N = 548 pregnant mothers with mobile phones		Weekly SMS reminders of ANC visits and pregnancy health information; two-way messaging for questions. Control = general health messages		Increase in mothers with 4 ANC visits and FBD		Difference-in-differences for FBD compared with prior pregnancies showed significantly greater increase in Intervention group (29% vs 13%).	
Bangal et al, 2017 [24]	RCT in Ahmednagar, India. N = 400 pregnant mothers with mobile phones.		Phone call reminders for ANC visits. SMS messages with pregnancy health information.		Increase in mothers with 4 ANC visits, FBD and post-natal checks		>4 ANC: 57.5% (I) vs 23.5% (C) (*P* < 0.0001). FBD: 91.5% (I) vs 89% (C) (*P*-value not given). No postnatal visits: 5.9% (I) vs 29.3% (C) (*P* < 0.001).	
Odeny et al, 2014 [25]	RCT in Nyanza region, Kenya; N = 388 HIV+ pregnant mothers enrolled in a prevention of mother-to-child transmission of HIV project, randomized to Intervention (n = 195) or Control (n = 193)		Intervention: 8 prenatal and 6 postnatal SMS messages. Participants in both study arms had SMS access to a study nurse.		Increase in postnatal care attendance and infant HIV testing by 8 weeks postpartum		Results (I. vs C.): Postnatal care attendance: 19.6% vs 11.8% (RR = 1.66; 95% CI = 1.02-2.70). Infant HIV testing: 92.0% vs 85.1% (RR = 1.08; 95% CI = 1.00-1.16)	
Uddin et al, 2016 [26]	Quasi-experimental pre/post evaluation using population level household surveys in Bangladesh; N = 4158		SMS reminders to mothers timed to vaccination schedule		Increase vaccination coverage in hard-to-reach groups		Difference-in-differences for full vaccination: +29.5% (rural; *P* < 0.001) & +27.1% (urban; *P* < 0.05). OR = for rural: 3.8 (95% CI = 1.5-9.2); OR = for urban: 3.0 (95% CI = 1.4-6.4)	
Haji et al, 2016 [27]	Quasi-experimental evaluation in 3 districts of rural Kenya with low coverage of 3rd dose pentavalent vaccine; N = 1116 children receiving first dose pentavalent vaccine		SMS vaccination appointment reminders compared with stickers or standard of care		Increase full vaccination coverage		SMS group significantly less likely to miss 3rd vaccine dose (OR = 0.2, CI = 0.04-0.8)	
Gibson et al, 2017 [28]	Cluster RCT in rural Kenya; N = 2018 infants from 152 villages		SMS reminders before pentavalent & measles vaccination dates. Parents in 2 groups also received incentives of either KES75 (US$0.88) or KES200 (US$2.35) for timely immunization.		Increase full vaccination coverage (BCG, measles, 3 doses polio, 3 doses pentavalent) at 12 months		Overall 86% fully immunized. No significant effects for SMS or SMS+KES75 groups. SMS+KES200 group were significantly more likely to achieve full immunization vs control (RR = 1.09; 95% CI = 1.02-1.16; *P* = 0.014)	
Zhou et al, 2016 [29]	Cluster RCT in 351 villages in Shaanxi Province, China; N = 1818 infants age 6-12 mo.		Free delivery of micronutrient powders plus daily SMS usage reminders compared with free delivery only and control		Increase caregiver compliance with micronutrient powder regimen; reduce child anaemia.		Higher compliance in SMS group (marginal effect: 0.10; 95% CI = 0.03-0.16). Greater decrease in anemia in SMS group relative to control group (marginal effect: -0.07; 95% CI = -0.12, -0.01), but not relative to delivery-only group (marginal effect = -0.03; 95% CI = -0.09-0.03).	
Alam et al, 2017 [30]	Observational study comparing Aponjon subscribers from 5 districts of Bangladesh who did and did not receive prenatal messages; N = 476		Twice-weekly maternal and newborn health messages by SMS or IVR		Increase in skilled birth attendance; early initiation of breastfeeding; delayed newborn bathing; and postnatal care visits.		No significant differences between groups for any outcome.	
Coleman et al, 2017 [31]	Retrospective study of HIV+ pregnant mothers, South Africa; N = 235 mothers receiving SMS intervention compared with non-users (N = 586)		Twice-weekly maternal health SMS messages tailored to stage of pregnancy and first year postpartum		Increase in ANC attendance and timely infant HIV testing; reduce low birth weight (<2500g).		SMS group were significantly more likely to attend ≥4 ANC visits (RR = 1.41; 95% CI = 1.15-1.72) and less likely to have a low birth weight infant (RR = 0.14; 95% CI = 0.02-1.07).	
**Intervention studies – grey literature:**								
Chowdhury, 2015 [32]	Retrospective observational study of MAMA’s Aponjon project in Bangladesh; N = 1473 (600 users +873 non-users matched by propensity score)		Subscriber-based MNCH messaging service using SMS or IVR, tailored to stage of pregnancy and infancy		Increase care seeking for MNCH		8/19 maternal care practices and 1/12 neonatal care practices significantly associated with Aponjon use (*P* < 0.05). No effect on infant feeding indicators.	
Coleman & Xiong, 2017 [33]	Retrospective case-control study using clinical records, comparing MomConnect subscribers matched with non-subscribers in Johannesburg, South Africa. N = 98 per group for ANC data; n = 33 per group for infant immunization data.		Subscriber-based messaging service delivering twice-weekly pregnancy and post-partum health messages to personal phones; help desk for registered users to ask questions and give feedback on health services		Primary: ≥4 ANC visits; Secondary: total ANC visits; infant birth weight; infant immunization coverage		Descriptive results (I. vs C.) as sample size not achieved. ≥4 ANC visits: 68.4% vs 70.4%. Full immunization at 1 y: 97% vs 93.9%. 80.5% MomConnect users were satisfied with the service.	
MatCH, 2016 [34]	Mixed methods evaluation of MomConnect (2011-2014) in KwaZuluNatal, South Africa. Quantitative: analysis of project monitoring data. Qualitative: interviews with subscribers (n = 60) and health workers (n = 37)		Subscriber-based messaging service delivering twice-weekly pregnancy and post-partum health messages to personal phones, linked to electronic medical records		Improved maternal and infant health and uptake of Prevention of Mother-to-Child HIV Transmission services		Enrollment targets were achieved and subscribers were highly satisfied with the SMS service. Further training for health workers and improved connectivity at health facilities were recommended to fully integrate the SMS service with electronic medical records.	
Healthbridge Foundation of Canada, 2016 [35]	Quasi-experimental mixed methods study of 3M project (Men using Mobile phones to improve Maternal health) in Jharkhand state, India; N = 207 couples with pregnant mothers, divided into Intervention (n = 104) and Control groups (n = 103). Qualitative data collected through stakeholder interviews and six focus group discussions with participants.		Husbands in intervention group received weekly IVR messages with maternal health information and reminders. Mothers in both groups were counseled by frontline health workers using a job aid app with multimedia messages.		Improved uptake of maternal health services (4+ ANC visits; consumption of 100 iron-folic acid tablets; FBD)		On average, husbands in Intervention group listened to 9 of 29 messages, with 31% listening to none. Results (I. vs C.):4+ ANC visits: 68.3% vs 58.3%; *P* > 0.05. 100 Iron-folic acid tablets: 33.7% vs 27.2%; *P* > 0.05. FBD: 93.3% vs 62.1%; *P* < 0.001.	
**Implementation studies – published literature:**								
Crawford et al, 2014 [36]	Analysis of electronic monitoring records and quarterly phone-based user surveys in Chipatala Cha Pa Foni project, Malawi		Subscriber-based MNCH messaging service delivered through SMS or IVR sent to personal phone, or IVR stored and retrieved from a community phone		Increase knowledge and coverage of home- and facility-based MNCH care		Message delivery success was greatest for SMS subscribers (30% of users) who were also more likely to report intended or actual behavior change (*P* = 0.01)	
Jiang et al, 2018 [37]	Description of implementation process and summary of process evaluation findings from monitoring records and qualitative interviews with participants at midterm (n = 22) and endline (n = 15).		Weekly SMS on infant feeding from 3rd trimester to 12 months postpartum; participants could text questions to a health professional		Increased EBF duration		3-phase implementation process: formative study; baseline questionnaire; message bank development. Process evaluation found high acceptability but preference for more in-depth and personalized content.	
Flax et al, 2016 [38]	Evaluation of feasibility and acceptability of using group cell phones in cluster RCT with micro-credit clients in Nigeria. Analysis of data from exit interviews (n = 195) and in-depth interviews (n = 17) with participants, and focus group discussions (n = 16) with non-participants		Intervention x10 months: monthly large group breastfeeding education; weekly SMS to small groups who presented to monthly large group		Increase timely breastfeeding initiation and EBF duration		Participants reported that the group cell phones worked well (64%) and 44% met at least weekly to share BCC messages. Participants in groups meeting at least weekly were more likely to practice EBF to six months than those in groups that never met (OR = 5.6; 95% CI = 1.6-19.7).	
Entsieh et al, 2015 [39]	Qualitative study; in-depth interviews (n=19) and focus group discussions (n=25 participants) with mothers who used Mobile Midwife messaging service in Ghana		Subscriber-based messaging service for maternal health		Improve maternal health and care-seeking		Participants described a gradual process of gaining trust in the Mobile Midwife messages, but needing to balance the new content with traditional practices. Engagement with the messages increased awareness of the need for skilled care and birth preparedness.	
LeFevre et al, 2018 [40]	Evaluation of MomConnect reach and exposure from August 2014-April 2017 using system-generated data, South Africa		Subscriber-based messaging service delivering twice-weekly pregnancy and post-partum health messages to personal phones; help desk for registered users to ask questions and give feedback on health services		Improve maternal health and quality of health care services.		Half of all women attending ANC-1 registered with MomConnect (n = 1 159 431) and subscribers received over 80% of messages sent. In 2016, 26% of attempted registrations failed, indicating a need for on-going system monitoring and improvement.	
Skinner et al, 2018 [41]	Qualitative study; in-depth interviews (n = 32) and 7 focus groups with MomConnect users, South Africa		Subscriber-based messaging service delivering twice-weekly pregnancy and post-partum health messages to personal phones; help desk for registered users to ask questions and give feedback on health services		Improve maternal health and quality of health care services.		Participants were satisfied with both the content and delivery system, with many saving the messages for future reference. Awareness of the help desk service was low.	
Xiong et al, 2018 [42]	Evaluation of system data collected from August 14, 2014 to March 31, 2017 for MomConnect helpdesk, South Africa		Subscriber-based messaging service delivering twice-weekly pregnancy and post-partum health messages to personal phones; help desk for registered users to ask questions and give feedback on health services		Improve maternal health and quality of health care services.		Approximately 8% of MomConnect subscribers used the helpdesk (n = 95.288), sending over 250 messages per day; 78.5% were health questions.	
Huggins & Valverde, 2018 [43]	Systems theory analysis of mNutrition using Malawi as a case study		SMS service delivering MNCH, household nutrition and agriculture content to smallholder farmers		Improve maternal and child nutrition		mNutrition was implemented within a complex system. Limited integration between sub-systems promoted more rapid implementation but likely compromised effectiveness and sustainability of messaging.	
**Implementation study – grey literature:**								
Chakraborty et al, 2019 [44]	Descriptive summary of monitoring findings (user surveys and focus group discussions) in Bihar, India		JEEViKA Mobile Vaani: interactive IVR platform allowing users to listen to pre-recorded content and record their own messages (curated). Core content focused on maternal diet diversity, complementary feeding, diarrhea management and social entitlements.		Increase intra-household dialogue on maternal and child nutrition, leading to improved practices		Extensive training facilitated user adoption of the JeeViKA platform. Implementing through self-help groups increased women’s participation but many were older women who engaged less with core content. Including non-core topics of interest to users increased overall engagement with content.	
**Feasibility studies – published literature:**								
Datta et al, 2014 [45]	Pre/post knowledge test and qualitative assessment in Tamil Nadu, India; N = 120		Participants received 10 MNCH messages over 10 d via SMS		Assess feasibility of SMS for improving MNCH knowledge		Knowledge scores improved and qualitative data indicated acceptability of SMS	
Hazra et al, 2018 [46]	Quasi-experimental study in rural Uttar Pradesh, India. Quantitative survey (N = 881 husbands & 956 women). Qualitative in-depth interviews with 10 couples and 2 focus group discussions with frontline health workers		Husbands of pregnant women received IVR messages on 5 MNCH topics twice-weekly over 4 months		Assess feasibility of IVR to husbands for improving household MNCH discussions and practices		34% participants reported receiving messages; 16% discussed messages at home. Main barrier: calls came while at work. Mothers with husbands who discussed messages were more likely to report ANC visit in 3rd trimester (OR = 1.72, *P* < 0.05), postnatal visit within 7 d (OR = 3.02, *P* < 0.05) & delayed newborn bathing (OR = 1.93, *P* < 0.05).	
Huang & Li, 2017 [47]	Survey of 129 randomly selected mothers who had registered for IVR service through community midwives in Cambodia.		Seven messages on neonatal care sent to mother’s phone from postnatal day 3-28		Assess acceptability of IVR to improve newborn care		Intervention was well accepted, with 60% indicating willingness to pay for the service. 43% reported taking baby to the health centre because of the IVR messages.	
McBride et al, 2018 [48]	Qualitative endline evaluation of mMom users, Vietnam. 2 Focus Groups and 8 interviews with frontline health workers; 4 focus group discussions and 30 interviews with participants (n = 60)		2-3 times weekly SMS during pregnancy & infancy. 15% interactive messages. SMS service linked to Health Management Information System to enhance contact and follow up by health providers.		Increase access to MNCH services by ethnic minority women in remote areas		High satisfaction with SMS and most expressed willingness to pay for the service. Participants reported increased knowledge and care seeking and stronger relationships with frontline health workers.	
Prieto et al, 2016 [49]	Mixed methods study: quantitative survey (all) and content analysis of text messages (groups 2 & 3). N = 78 Spanish speaking pregnant women or mothers of young infants in Guatemala.		All participants received a free basic mobile phone with prepaid credit. Group 1: 2 × weekly breastfeeding SMS. Group 2: group text messaging to discuss MNCH topics. Group 3: same as group 2 + breastfeeding messages + health professional facilitated group. Group 4: control		Compare text messaging approaches for MNCH with focus on breastfeeding		Knowledge of EBF at endline was greater in Groups 1 (60%) & 3 (50%) than Groups 2 (8%) & 4 (25%). Content analysis (groups 2 & 3): 62% messages related to social support; 35% health info; 3% other	
Domek et al, 2016 [50]	Pilot RCT at 2 public health clinics in Guatemala City. N = 321 infants age 8-14 weeks presenting for 1st immunization dose.		3 SMS reminders one week before 2nd and 3rd immunization dose.		Assess feasibility and acceptability of SMS to improve adherence to full immunization.		No significant differences between groups (i. vs C.) for dose 2 (95.0% vs 90.1%; *P* = 0.12) or dose 3 (84.4% vs 80.7%; *P* = 0.69). Over 90% of participants wanted future SMS reminders with the Intervention group more willing to pay for the service (67.5% vs 49.6%; *P* = 0.01).	
Wakadha et al, 2013 [51]	Pilot study in western Kenya; N = 72 mothers of infants age 0-3 weeks		SMS reminders sent 3 d before and on the day of Pentavalent vaccine doses 1 and 2. Mothers of infants vaccinated within 4 weeks of the scheduled date received KES150 (US$2) as either airtime credit (1/3) or mobile money transfer (2/3).		Assess feasibility and acceptability of SMS plus conditional cash transfers to improve timely vaccination.		63 children had known vaccination status at endline, of whom 90% received dose 1 and 85% received dose 2 within 4 weeks of the scheduled date. All mothers preferred mobile money transfer to airtime credit.	
**Formative research – published literature:**								
Hmone et al, 2016 [52]	Qualitative formative study with pregnant mothers and family members (n = 20 in-depth interviews) and service providers (n = 7 interviews; n = 15 focus group participants) in Yangon, Myanmar		SMS to increase EBF		Identify barriers and facilitators of EBF and of SMS communication in order to guide the design and messaging content for an RCT		EBF barriers include grandmothers’ recommendations for early supplementation, perception of insufficient breastmilk, and mothers’ return to work. Contextualized messages sent in the evening were recommended for the intervention.	
Weerasinghe et al, 2016 [553]	Qualitative study in 2 tea estates, Sri Lanka. Focus group discussions with mothers (n = 109), fathers (n = 30) and older women (n = 32). Interviews with health and childcare providers (n = 15).		SMS or IVR for infant and young child feeding counseling		Explore issues related to infant and young child feeding, use of mobile phones and sources of nutritional information.		Most household have mobile phones, primarily used by men for voice calls. Infant and young child feeding counseling is provided in-person at regular growth monitoring sessions. Mothers and health workers preferred in-person counseling but were open to IVR as a supplementary intervention.	
Kazi et al, 2017 [54]	Survey conducted in 8 health facilities in Northern Kenya; N = 284 attendees at routine ANC and immunization clinics.		SMS to promote ANC and immunization		Explore potential for SMS delivery platform		88% had access to mobile phones; 92% of those were interested in receiving a weekly SMS health message.	
Yamin et al, 2018 [55]	Cross-sectional survey in Nangarhar Province, Afghanistan; N = 240 women		Direct Messaging for MNCH, including ANC and immunization reminders.		Explore perceptions related to use of mobile phones for MNCH communication.		91.7% routinely used mobile phones. 87.1% were willing to receive health messages. IVR was preferred to SMS.	
Calderon et al, 2015 [56]	Cross-sectional survey in Arequipa, Peru. N = 220 mothers with at least one child under 5 y old		2-way SMS for child care during illness		Explore feasibility and acceptability of 2-way SMS platform		95% reported mobile phone access. 86% were interested in using SMS to receive child health messages or seek care but only 27% wanted to receive appointment reminders.	
Brinkel et al, 2017 [57]	Qualitative study in four districts of the Greater Accra Region, Ghana; 4 focus group discussions with caregivers of at least one child under 10 y old N = 40		IVR for health seeking		Explore feasibility and acceptability of IVR platform		Participants were open to IVR but had no prior experience with it. Social, infrastructure and technology literacy barriers were identified. A toll-free number and training was recommended.	
**Case studies – grey literature:**								
MAMA Bangladesh [58]	Narrative report of intervention design process based on formative research findings		Subscriber-based MNCH messaging service using SMS or IVR; hotline for subscribers to contact a female doctor		Key design elements identified through formative research: options for users with low technology literacy; inclusion of family members; use female doctor voice; timing of message delivery; record messages in local dialects; adapt message content & frequency for rural vs urban clients			
MOTECH ‘Mobile Midwife’ [59]	Narrative report of intervention design process and lessons learned		Subscriber-based IVR service for maternal health in rural Ghana		Message design & delivery guided by formative research. Collaboration with respected partners increased trust, and mHealth initiative integrated with efforts to improve ANC services.			
Mobile Information for Maternal Health [60]	Narrative report of project design and monitoring results		Subscriber-based MNCH messaging service using IVR or SMS for pregnant mothers in Ghana, with interactive messages to assess retention of content		5400 subscribers; 73% report the messages are useful and the majority listen to >80% of the messages. Monitoring data showed IVR messages of 90 s retained 70% of users, so content was redeveloped to fit this optimal timing.			
‘Healthy pregnancy, healthy Baby’ [61]	Narrative report of intervention design with lessons learned		Subscriber-based text messaging service in Tanzania with tracks for pregnant mothers, their supporters and general information-seekers		Lessons learned on content development: messages must be localized & pre-tested; best to craft in local language, not translate; include fun messages with formal health content. Implementation challenges include low female phone ownership & literacy; poor connectivity; responding to questions from subscribers.			
‘Healthy Pregnancy Healthy Baby’ [62]	Narrative report of lessons learned from GSMA engagement with the ‘Healthy Pregnancy Healthy Baby’ program from 2014-2017, based on user feedback and surveys		Subscriber-based SMS service in Tanzania offering content on MNCH, prevention of mother-child HIV transmission and family planning, embedded in Ministry of Health mHealth platform		Key lessons learned: self-registration is challenging for many users but frontline workers assisting registration need regular refresher training; promoting the program via radio and TV ads doubled registrations; in-kind support from 4 national mobile network operators allows large reach at no cost to users; content must be both accurate and contextually appealing; appointment reminders are highly valued.			
People In Need Cambodia [63]	Narrative report of intervention design process and pilot study		Subscriber-based IVR service in Cambodia delivering seven messages on neonatal care to mother’s phone from postnatal day 3-28		Message design and delivery guided by formative research. Contextual adaptations included use of voices representing locally authoritative figures; content geared to build community support for improved newborn care; midwives registered mothers after delivery; subscribers also received a hanging mobile toy with the messages. Key partnerships supported both content development and technology systems.			
Kilkari [64]	Narrative report of lessons learned and best practices for implementation at scale		Subscriber-based weekly IVR service for MNCH in six states of India		2 million subscribers in first 12 months; 42% listen to ≥75% of messages. Content is narrated by a female doctor character and targets both fathers and mothers. Call costs are covered by Government of India. Ongoing investment in skilled technology support is needed to maintain large-scale implementation.			
mNutrition [65]	Narrative report of intervention design and monitoring results		SMS service delivering MNCH and nutrition content in 8 countries: Malawi, Ghana, Tanzania, Kenya, Nigeria, Zambia, Uganda, Mozambique		Multi-partner initiative delivering localized content through mobile network providers. 1.59 million users across 8 countries by December 2017. 69% of users reported correct nutrition knowledge and practices vs 57% and 56% of non-users. 42% of users report sharing content with others. Repeated messages are appreciated and reinforce key content.			

EBF – exclusive breastfeeding, FBD – facility-based delivery, RCT – randomized controlled trial, ANC – antenatal care, IVR – interactive voice response, MNCH – maternal newborn and child health

**Table 3:** Voice counseling studies

Study	Design	Voice counseling intervention	Target outcomes	Key results
**Intervention studies – published literature:**				
Maslowsky et al, 2016 [66]	Prospective evaluation in Quito, Ecuador; N = 178 Spanish speaking mothers recruited as inpatients at delivery and randomized to intervention (n = 102) or control (n = 76).	Structured postnatal education via mobile phone within 48 h of delivery; phone access to nurse during business hours for newborn’s first 30 d	Improve postnatal care and maternal and infant health	EBF at 3 mo: 86.7% (I.) vs 66.7% (C.); *P* = 0.005. Neonatal well baby check attendance: 72% (I.) vs 53.3% (C.); *P* = 0.022. No significant differences for 2-months well baby visit attendance or contraception use.
**Formative and qualitative studies – published literature:**				
Huq et al, 2014 [67]	Qualitative pre/post study in rural Bangladesh. Pre: interviews with Community Skilled Birth Attendants (CSBA) (n = 12) & mothers (n = 14). Post: in-depth interviews (n = 6 CSBA); semi-structured interviews (n = 27 CSBA). 1 FGD with 10 mothers.	‘mobile pathways’ using toll free numbers: mother/family calls CSBA who provides advice and/or consults with experts	Increase access to prompt, quality care for complications during pregnancy & delivery	Participants perceived improvement in timely access to care, access to specialist care and care seeking.
Khan et al, 2018 [68]	Formative study in 2 sub-districts, Bangladesh. In-depth interviews with mothers (n = 24) and health workers (n = 13); focus group discussions with fathers (n = 4) and grandmothers (n = 4).	Mobile phones for infant and young child feeding counseling	Increase quality and coverage of counseling by frontline health workers (FLWs)	Voice calls were preferred by both mothers and FLWs, but issues of phone access (mothers), cost (FLWs) and building trust with household decision-makers were identified.
**Intervention studies – grey literature:**				
Sellen et al, 2013 [15]	Randomized control trial in Kenya; n = 752 HIV-mothers randomized to two intervention groups or control (standard of care)	Group 1: Proactive bi-weekly call from same counselor to 3 months post-partum; unlimited access to text & phone support. Group 2: monthly peer support group facilitated by infant and young child feeding counselor	Increased prevalence of EBF at 3 months postpartum	EBF at 7 d: 94% all groups. EBF at 3 months: 90.9% (group 1), 82.8% (group 2), 78.2% (control) [*P* = 0.0017 between group 1 and other 2 groups, non-significant between group 2 and control]

EBF – exclusive breastfeeding, CSBA – Community Skilled Birth Attendant, FLWs – frontline health workers

**Table 4:** Studies of direct messaging + voice counseling

Study	Design		Intervention	Target outcomes	Key results	
**Intervention studies – published literature:**						
Fotso, Robinson et al, 2015 [69]	Two-arm, pre/post quasi-experimental effectiveness evaluation using cross-sectional household surveys; n = 2810 (baseline); n = 3643 (endline)		Chipatala Cha Pa Foni project, Balaka district, Malawi. Intervention: subscriber-based ‘tips and reminders’ weekly messaging service and case management hotline for health advice and referrals; community-shared phones hosted by volunteers to increase access	Increased knowledge and use of home- and facility-based MNCH care	Intention-to-treat analysis: negative effects on child care practices; no effect on knowledge or maternal care. Treatment-on-treated effect analysis: positive effects on home-based (*P* < 0.01) and facility-based (*P* < 0.05) maternal care, and home-based child care (*P* < 0.01).	
Fotso, Bellhouse et al, 2015 [70]	As above		As above	As above	Home-based care for child health improved through increased bed net usage (*P* < 0.01); facility-based care seeking for child fever decreased (*P* < 0.01)	
Patel et al, 2018 [71]	Two-arm cluster randomized trial of mothers (n = 1036) recruited through baby-friendly hospital antenatal care facilities; 2 clusters per arm		Daily SMS, weekly call from certified lactation counselor, and phone number to call counselor from 3^rd^ trimester to 6 months postpartum.	Improve exclusive breastfeeding (EBF)	EBF (I. vs C.): Birth: 74.3 vs 73.7%; Follow up: (*P* < 0.001) 6 weeks: 97% vs 80.9%; 10 weeks: 97.6% vs 78.1%; 14 weeks: 96.2% vs 70.7%; 6 months: 97.3% vs 48.5%	
**Intervention studies – grey literature:**						
Watkins et al, 2013 [72]	Mixed methods evaluation. Quantitative: Two-arm, pre/post quasi-experimental design; cross-sectional population-based household surveys; n=2810 (baseline); n=3643 (endline). Qualitative: Key Informant Interviews (n=47) with health staff, village leaders & project staff; Focus Group Discussions (n=12 with 10 participants each) with users, non-users and mothers who had not heard of the project; in-depth interviews (n=16) with 1 participant per focus group and her husband; hearsay ethnographic journals (n=46)		Chipatala Cha Pa Foni project, Balaka district, Malawi Intervention: subscriber-based ‘tips and reminders’ weekly messaging service + case management hotline providing protocol-based advice and referrals; community-shared phones hosted by volunteers to increase access	Increased knowledge and use of home- and facility-based MNCH care	Intervention reached 19% of eligible mothers; 61% used the community phone to access services. Endline survey: no significant differences in knowledge or home-based care practices; negative effect on uptake of facility-based services for child health. Qualitative: high user satisfaction and perceived improvements in quality of facility-based care. Non-users faced technical and socio-cultural barriers.	
Health Alliance International, Liga Inan Technical Briefs 2 & 3 [73,74]	Pre/post intervention study comparing intervention and control communities in Timor-Leste; N=603 mothers of children under 2 years at endline (breakdown between Intervention and Control areas not provided)		Registered pregnant mothers receive twice weekly SMS education and reminder messages and can request a call from their midwife. Midwives call all clients 3 weeks before due date.	Improve uptake of maternal and neonatal health services, particularly antenatal care (ANC), facility-based delivery, skilled birth attendance, postpartum care for mother and postnatal care for infant within 2 days	Changes from baseline to endline (I. vs. C.; *P*-values not given): 4+ ANC visits: 76% to 85% vs. 67% to 81%; Facility-based delivery: 32% to 49% vs. 29% to 28%; Skilled birth attendance:48% to 62% vs. 38% to 36%; Postpartum care within 2 days: 26% to 51% vs. 38% to 25%; Postnatal care within 2 days: 20% to 39% vs. 32% to 22%	
**Implementation studies – published literature:**						
Larsen-Cooper et al, 2015 [75]	Mixed-methods study of use of community shared phones; data from monthly usage records and larger program evaluation qualitative data		Chipatala Cha Pa Foni (CCPF) project, Balaka district, Malawi. Intervention: subscriber-based ‘tips and reminders’ weekly messaging service + case management hotline providing protocol-based advice and referrals; community-shared phones hosted by volunteers to increase access.	Increased knowledge and use of home- and facility-based MNCH care	9328 users called the hotline; 5884 subscribed to messaging service. 68% accessed services through non-owned phone, but usage decreased over time.	
Larsen-Cooper et al, 2016 [76]	Cost-outcome analysis of CCPF project, Malawi, using a base cost model to estimate costs per user and per contact. Costs were mapped to MNCH indicators which showed improvements in the pre/post evaluation in order to estimate costs of health improvements, with sensitivity analysis to explore effects of higher intervention usage.		Chipatala Cha Pa Foni (CCPF) project, Balaka district, Malawi. Intervention: subscriber-based ‘tips and reminders’ weekly messaging service + case management hotline providing protocol-based advice and referrals; community-shared phones hosted by volunteers to increase access.	Increased knowledge and use of home- and facility-based MNCH care	9798 unique users accessed CCPF, at a cost of US$29.33 per user and US$4.33 per successful contact (message sent or received). Costs ranged from US$67 to US$355 for each pregnant user reporting improvements in MNCH knowledge, and from US$128 to US$256 for each user reporting changes in MNCH practices. Sensitivity analysis showed a 48% reduction in cost per user if the system operated at full capacity.	
**Implementation study – grey literature:**						
Health Alliance International, Liga Inan Technical Brief 1 [77]	Analysis of participation data from Liga Inan endline evaluation,Timor-Leste; N = 603 mothers with children under 2 years of age (breakdown between Intervention and Control areas not provided)		Registered pregnant mothers receive twice weekly SMS education and reminder messages and can request a call from their midwife. Midwives call all clients 3 weeks before due date.	Improve uptake of maternal and neonatal health services	70% of pregnant women in Intervention area participated, 82% using their own phone. 97% found the messages easy to understand and 53% reported sharing messages with another person.	
**Feasibility studies – published literature:**						
Huda et al, 2018 [78]		Mixed methods pilot study with pre/post quantitative surveys and post-intervention qualitative interviews (n = 21). N = 340 pregnant and lactating mothers in rural Bangladesh.	2 × weekly IVR messages tailored to stage of pregnancy/infancy, bi-weekly nutrition counseling calls, call centre access and monthly unconditional cash transfers of BDT787 (US$10).	Improve perceptions of maternal and infant nutrition		96% of participants were satisfied with the IVR messaging content and frequency. 81% of call centre contacts were successful, and 50.9% of participants phoned the call centre at least once. Preferences for nutrition advice: 62.2% IVR + voice counseling; 32.7% voice counseling; 5.1% IVR.
Alhaidari et al, 2017 [79]		Pilot randomized controlled trial in antenatal clinics affiliated with a maternity hospital in Iraq; N = 250 pregnant women.	Weekly text messages tailored to stage of pregnancy. Hotline for pregnancy questions.	Assess feasibility and acceptability of text messaging to improve uptake of ANC services.		Median number of ANC visits was higher in intervention group (4 vs 2; *P* < 0.001). 60.8% of I-group made ≥1 hotline call, for a total of 314 calls. 90.7% would recommend the SMS service.
**Formative study – published literature:**						
Jennings et al, 2013 [80]	4 in-depth interviews with nurses and 6 focus groups (2 each with HIV+ women, male partners & FLWs) in Nyanza, Kenya; N = 45, recruited through 2 hospital PMTCT programs		n/a	Assess perceptions of cell phone communication to support PMTCT service delivery	Preferred mHealth platform combines SMS for reminders and basic information with voice counseling for discussion; health workers prefer in-person sessions	
**Formative study – grey literature:**						
Napier & Peterson, 2013 [81]	Doer/non-doer analysis of qualitative interviews with adolescent mothers in Honduras; n = 31		Initial intervention: monthly breastfeeding support groups, weekly SMS and additional voice counseling support	Improved breastfeeding practices among adolescent mothers	Identified technical and social barriers to cell phone support	
**Programmatic case study – grey literature:**						
Health Alliance International [82]	Narrative description of intervention design, monitoring and lessons learned from the Liga Inan mHealth program in Timor-Leste.		Registered pregnant mothers receive twice weekly SMS education and reminder messages and can request a call from their midwife. Midwives call all clients 3 weeks before due date.	Integration with Ministry of Health facilitates participation and sustainability. Telephone surveys indicate high user satisfaction with messages and a high rate of sharing content with family members. Participants value the enhanced communication with their midwife. In-depth interviews with midwives found high satisfaction with the service despite increased workload. Variations in access to health services affects uptake of recommended practices.		

MNCH – maternal, newborn and child health, EBF – exclusive breastfeeding, ANC – antenatal care, IVR – interactive voice response, PMTCT – prevention of mother-to-child transmission of HIV

**Table 5:** Job aid application studies and reports

Study	Design	Intervention	Target outcomes	Key results
**Intervention studies – published literature:**				
Martinez-Fernandez et al, 2015 [83]	Retrospective observational study of population level baseline to endline changes in mortality comparing intervention communities (n = 125) with non-intervention areas in rural Guatemala	FLWs equipped with cell phone-based tools, including job aid app, distance learning modules, and access to supervisors for phone consultation.	Maternal mortality, Infant mortality	Maternal mortality rate decreased in intervention areas (309 to 254) but increased in control areas (338 to 558) (*P* < 0.05 between groups). Infant mortality rate decreased in both groups (25-13; 27-20) (*P* = 0.054 between groups).
McNabb et al, 2015 [84]	Pre/post evaluation of pregnant mothers (n = 267) in Nigeria; baseline data collection at first ANC visit, endline 12 months later	Job aid app to guide facility-based health workers through ANC protocols and track client data in real time; 13 BCC audio files embedded	Improve quality of ANC care and client satisfaction	Quality score increased from 13.33 (baseline) to 17.15 out of 25 (*P* < 0.0001); greatest improvements in BCC message delivery
Battle et al, 2015 [85]	Mixed methods evaluation in Zanzibar, Tanzania. Quantitative: system generated monitoring data; N = 13 231 registered mothers who gave birth in the project period. Qualitative: semi-structured interviews with mothers (n = 27), FLWs (n = 25) and health facility staff (n = 12)	Job aid app supporting safe deliveries through client registration, monitoring, BCC, communication with health facilities and money transfer for transport	Increase uptake of facility-based delivery (FBD) and postnatal care	FBD: 75% vs 35% in most recent Demographic & Health Survey. Postnatal care attendance: 88%. Interview findings: referral and communication functions increased FLW confidence; frequent contact with FLWs and transport service increased FBD but cost of FBD was a barrier
Shiferaw et al, 2016 [86]	Non-randomized prospective controlled evaluation in 10 health facilities (5 intervention, 5 control) of Amhara region, Ethiopia. Exit interviews with mothers attending ANC: n = 933 (baseline); n = 1037 (endline). Chart review of ANC clients: n = 1224	Job aid app for facility-based health workers supporting client registration, ANC and postnatal care visit reminders, decision support, and pregnancy health information. Health workers contacted clients on receiving visit reminders.	Increase in mothers with 4 ANC visits, FBD and postnatal care at each facility	Results (I. vs C.): 4+ ANC visits: 27% vs 23.4% (AOR = 1.31; 95% CI = 1.00-1.72). FBD: 43.1% vs 28.4% (AOR = 1.98; 95% CI = 1.53-2.55). Postnatal care attendance: 41.2% vs 21.1% (AOR = 2.77; 95% CI = 2.12-3.61).
Hackett et al, 2018 [87]	Cluster randomized trial in 32 villages in rural Tanzania; N = 572 mothers selected for postnatal survey	Job aid app supporting FLWs with data management and real-time guidance for prenatal home visits	Increase uptake of FBD	FBD significantly more likely in I-group (74% vs 63%; OR = 1.96; 95% CI = 1.21-3.19; *P* = 0.01).
Ilozumba et al, 2018 [88]	Quasi-experimental study in 3 sub-districts of rural Jharkhand, India; N = 2200 mothers with infants <12 months old	2 sub-districts received NGO-led MNCH activities. Mobile for Mothers (MfM) app was added in 1 sub-district to support FLW home visits with data management and embedded multimedia BCC messages	Improve maternal health knowledge, ANC attendance and FBD	Mothers in the MfM group were significantly more likely than NGO or control groups to attend ≥4 ANC visits (OR = 1.23; 95% CI = 1.17-1.29; OR = 1.36; 95% CI = 1.30-1.42) and to have FBD (OR = 1.19; 95% CI = 1.13-1.25; OR = 1.34; 95% CI = 1.28-1.41)
Prinja et al, 2017 [89]	Pre/post quasi-experimental study in rural Uttar Pradesh, India. Annual Health Survey data served as pretest; matched with posttest household survey sample (N = 3106 mothers equally divided between intervention & control areas).	Job aid app supporting FLWs with data management and real-time guidance for home visits during pregnancy and infancy (ReMiND project)	Improve uptake of MNCH services	Large increases in both groups for 4 of 8 MNCH indicators. Significantly greater increases in the Intervention group for self-reporting illness during pregnancy (13.2 p.p.; *P* = 0.04) and after delivery (19.5 p.p.; *P* = 0.01)
**Intervention studies – grey literature:**				
Borkum et al, 2015 [90]	Cross-sectional survey in Bihar, India comparing mothers of infants <12 months old (n = 1550) in randomly selected intervention and control communities. Qualitative process evaluation: interviews with FLWs (n = 23) and project staff (n = 4)	Job aid app for home visits during pregnancy and infancy, with multimedia BCC messages embedded	Improve coverage and quality of FLW services	Intervention group more likely to receive home visits prenatally, in first week postnatal, and for complementary feeding (*P* < 0.05), and more likely to report 3+ antenatal care visits, birth preparedness, and timely initiation of both breastfeeding and complementary feeding (*P* < 0.05).
BBC Media Action, 2016 [91]	Observational study in Bihar, India, comparing mothers exposed to the Mobile Kunji intervention (n = 2543) vs unexposed (n = 956). Qualitative process evaluation: 4 focus groups and 28 in-depth interviews with FLWs	Counseling tool (Mobile Kunji) combining visual aid with IVR messages accessed through FLWs’ personal phones	Improve quality and engagement with BCC for key MNCH practices	Exposed mothers more likely to have saved the FLW’s phone number (OR = 2.72) and to have fed infants 6-11 months old from at least one food group in past 24 h (OR = 1.72) but no effect on family planning practices.
World Vision, 2018 [92]	Randomized controlled trial in Niger. N = 126 FLWs randomized equally to intervention and control groups were evaluated for their assessment of 544 sick children age 2-59 months	Job aid app supporting Integrated Community Case Management for childhood illnesses	Improved assessment of childhood danger signs and counseling for caregivers	Preliminary results show no significant difference between groups for identification of cough, diarrhea or fever. Mixed results for counseling. Overall no added benefit from the app for management of priority illnesses.
**Implementation studies – published literature:**				
Balakrishnan et al, 2016 [93]	Observational study comparing monitoring data from FLWs in intervention districts of Bihar, India with government statistics for the rest of the state and for the intervention district in the previous year	Job aid app for home visits during pregnancy and infancy, with multimedia BCC messages embedded	Improve quality, equity and efficiency of FLW delivery of 8 core MNCH services: pregnancy registration; registration in first trimester; 3 ANC visits; ≥1 tetanus toxoid vaccine; >90 iron folic acid tablets; delivery in health facility; early initiation of breastfeeding; and ≥1 postnatal home visit.	Coverage of all MNCH services was higher in implementation areas but statistical significance was not assessed. Utilization of MNCH services was similar between scheduled castes and others except for facility-based delivery. Instant data upload from app eliminated delay in data capture from paper forms.
Ilozumba et al, 2018 [94]	Mixed methods assessment of factors influencing outcomes of Mobile for Mothers (MfM) study in Jharkhand, India. Quantitative surveys with mothers (N = 740) and FLWs (n = 57) participating in the intervention. Qualitative interviews with FLWs (n = 28), mothers (n = 32) and men (n = 31).	Mobile for Mothers (MfM) app supporting FLW home visits with data management and embedded multimedia BCC messages	Improve maternal health knowledge, ANC attendance and FBD	MfM app increased FLWs’ knowledge, confidence and efficiency. Main barriers to ANC and FBD uptake were women’s workload, finances, household power dynamics and access to health services.
**Feasibility studies – published literature:**				
McConnell et al, 2016 [95]	Randomized trial in Kiambu county, Kenya. N = 104 postnatal mothers recruited from one private maternity hospital and individually randomized to home visit (n = 32), phone call (n = 41) and control (n = 31) groups	3-d postnatal checklist administered by FLWs either by mobile phone or home visit, compared with standard of care	Improve knowledge and care-seeking for postnatal danger signs	Postnatal checklist administered to 76% of phone group and 59% in home visit group. Infant care-seeking occurred earlier in home visit (2.0 d, *P* = 0.014) and phone call groups (1.8 d, *P* = 0.034) compared with control.
Amoah et al, 2016 [96]	Pilot study in 4 communities of rural Ghana; N = 323 pregnant women. Outcomes compared with preliminary survey (N = 100 women in project communities who were pregnant in past 5 years)	Job aid app supporting FLWs with registration, data management and point-of-care guidance for prenatal clients; ultrasound scans conducted in project communities for mothers unable to attend hospital.	Increase ANC attendance and FBD	40 births in study period; 30 (75%) had >3 ANC visits; 25 (62.5%) had FBD compared with 54% and 33% in the preliminary survey.
**Formative studies – published literature:**				
Modi et al, 2015 [97]	Descriptive report of design and feasibility testing of ImTeCHO app for FLWs (n = 45) in Gujarat, India. Feasibility study: Interviews with 6 FLWs and 2 medical officers; focus groups with 9 FLWs and 6 Auxiliary Nurse Midwives; home visit observations	Job aid app for FLWs with work flow scheduling, task monitoring, videos on key BCC messages; link to supervisory support for complex cases and ongoing monitoring	Improve coverage of key MNCH services assigned to FLWs	Job aid app well accepted & considered feasible but ongoing NGO support required for facilitation and technical support.
Kaphle et al, 2015 [98]	Demographics questionnaire completed by FLWs (n = 15); home visit observations (n = 14)	Job aid app for home visits	To develop methods to analyze 1) the effects of app adoption on the quality and experience of care; and 2) personal factors influencing app usage by FLWs	Quality scores were 33.4% higher for high users of the app (*P* = 0.04). No significant associations with individual factors were found.
**Qualitative studies – published literature:**				
Hackett et al, 2018 [99]	Qualitative study in rural Tanzania. In-depth interviews with FLWs (n = 60) and two rounds of focus group discussions with mothers (n = 56) participating in an mHealth trial	Job aid app supporting FLWs with data management and real-time guidance for prenatal home visits	Women’s reproductive health is a private matter in rural Tanzania, due to fear of exposure to witchcraft. FLWs are trusted confidants. FLWs believed smartphones enhanced data privacy but some mothers expressed concerns about data storage and who could access the phones.	
Pimmer & Mbvundula, 2018 [100]	Interpretive case study within the Millennium Village Project in rural Malawi. Focus group discussions with FLWs (n = 29) and interviews with supervisors (n = 3).	Job aid app for FLWs with embedded audio counseling messages related to health topics including MNCH and nutrition.	FLWs perceived the audio messages to support their work in three ways: i) legitimize the use of phones during home visits; ii) assist the FLW to deliver a comprehensive message; iii) support FLWs to persuade communities to adopt health practices.	
**Qualitative study – grey literature:**				
Treatman & Lesh, 2012 [101]	Interviews with implementers (n = 8) of CommCare app deployments in India	Job aid tool with embedded audio messages for BCC	Improve quality of counseling by FLWs	Audio messages improved FLW credibility and eased discussion of sensitive topics but design challenges with localization.
**Reviews & case studies – grey literature:**				
Chamberlain, 2014 [102]	Case study	Counseling tool (Mobile Kunji) combining visual aid with IVR messages in Bihar, India	User-centred design process: extensive formative research, prototype development; iterative testing & refinement. Partnerships built with 6 major mobile network operators who subsidize 90% cost of Mobile Kunji calls. >35 000 FLWs using the service with their own basic phones.	
Ramachandran, 2013 [103]	Case study	Cell phone-based audio-visual counseling tool for FLWs in Orissa, India	Iterative design process informed by qualitative data and concepts from theories of persuasion. Final tool used locally filmed video with built-in questions and pauses to encourage discussion.	
Manthan Project, 2013 [104]	Case study of mSAKHI app with summary findings from 2 pre/post quasi-experimental feasibility studies: i) self-learning and counseling (n = 86 FLWs); ii) postnatal care delivery (n = 57 FLWs)	Job aid app with multimedia functions	i) improved knowledge & counseling delivery by FLWs; ii) Improved coverage and quality of newborn care	i) greater frequency & completion of BCC message delivery (*P* < 0.05 for 6/9 messages); ii) intervention group more likely to correctly assess newborn health (*P* < 0.05 for 5 of 7 skills)
Chatfield & Javinski, 2015 [105]	Narrative review of CommCare evidence base, both published & grey literature (n = 40 papers)	CommCare job aid tool for FLWs	Documented evidence related to CommCare acceptability; contribution to improved access to care; quality, experience, and accountability of care; and changes in client knowledge & practices.	
Flaming et al, 2016 [106]	Narrative review of CommCare evidence base, both published & grey literature (n = 51 papers; 22 papers address MNCH)	CommCare job aid tool for FLWs	Descriptive review of evidence related to CommCare contribution to improvements in FLWs’ knowledge; performance and credibility; client knowledge and practices; and quality of care. Implementation challenges include technical difficulties, decrease in usage over time, and integrating data with local health systems.	
Keisling, 2014 [107]	Project case studies: i) pre/post evaluation (n = 206) with comparison group in Afghanistan; ii) supervisory app monitoring data in India	CommCare job aid apps for FLWs	Improved MNCH knowledge and practices	i) changes in 7 of 15 indicators including birth preparedness, ANC and FBD; ii) 136% increase in clients asking questions during home visits; 78% of mothers served by app gave birth in facilities
World Vision, 2015 [108]	Narrative overview of World Vision’s mHealth projects with country project examples from India, Indonesia, Sierra Leone & Uganda	MOTECH Suite with apps supporting five MNCH project models including home visits by FLWs	Strengthen community health systems	16 projects in 21 countries; 7 projects have >100 users. Implementation lessons: high acceptability of apps; need adequate technology training and support to FLWs
MIRA Channel, 2015 [109]	Project brief summarizing MIRA concept and results of pilot test with 50 FLWs	Mobile phone “channel” integrating health communication and management functions for rural women in India	Increase rural women’s access to health knowledge and care	59% increase in ANC attendance, 49% increase in FBD and 41% increase in immunization coverage with MIRA use
World Vision, 2018 [92]	Narrative description of evaluation and research findings related to World Vision’s mHealth deployments for FLWs in 11 countries	CommCare job aid apps for FLWs	Process evaluation surveys in Sierra Leone and Uganda found high acceptance of job aid apps, particularly by beneficiaries with greater exposure to mHealth. Formative research in Mauritania and Tanzania guided program adaptions related to technology, contextualization and integration with Ministry of Health systems. Analysis of projects in Uganda, Sierra Leone and India using the mHealth Assessment and Planning for Scale (MAPS) toolkit identified site-specific strengths to leverage and challenges to address in ongoing program plans.	

FLWs – frontline health workers, ANC – antenatal care; BCC – behaviour change communication, FBD – facility-based delivery, MNCH – maternal, newborn and child health, IVR – interactive voice response

**Table 6:** Interactive media study

Study	Design	Interactive media intervention	Target outcomes	Key results
**Intervention study – published literature**				
Santoso et al, 2017 [114]	Randomized controlled trial with pre/posttest design in Indonesia; N = 38 couples from antenatal service at 3 health centres randomized equally to intervention or control.	Android app for husbands to monitor pregnancy progress and receive information on danger signs, care and treatment. Both groups received counseling.	Improve husbands’ birth preparedness and complication readiness (BP/CR scores)	BP/CR score (I. vs C.): Pretest: 60.4 vs 61.5 (NS); 3 week posttest: 72.9 vs 62.6 (*P* = 0.001); Postpartum posttest: 81.8 vs 71.3 (*P* < 0.001)

### Direct messaging

Direct messaging delivers brief, standardized messages to users’ phones, using either short-message-service (SMS) for written text, or Interactive Voice Response (IVR) technology for audio content. This is the oldest form of BCC using mobile phones, and still widely used; we identified more documents related to direct messaging than the other three delivery approaches combined (**Table 1**). These documents describe pilot and intervention studies, implementation research, formative studies and programmatic case studies targeting a range of MNCH outcomes (**Table 2**).

Feasibility studies in a variety of settings have found direct messaging interventions to be acceptable to target users and to show potential for improving MNCH knowledge and practices. Datta et al. found high acceptance of SMS as a delivery channel for MNCH messages in rural India, and knowledge improved after participants received one message per day for ten days [45]. A quasi-experimental study in rural Uttar Pradesh, India, found that mothers whose husbands received and discussed IVR messages were more likely to visit the ANC in the third trimester and to have a postnatal visit within seven days (OR = 1.72; OR = 3.02; *P* < 0.05 for both) [46]. However, only 34% of participating husbands received the IVR messages as they could not accept the calls while at work [46]. A pilot study of an IVR service delivering seven messages within the first month postpartum in Cambodia reported high acceptability, with 60% of survey participants indicating willingness to pay for the service [47]. The mMom service in Vietnam aimed to improve ethnic minorities’ access to MNCH services, and was well received with participants reporting strengthened relationships with health workers [48]. In Guatemala, exclusive breastfeeding knowledge increased among mothers who received twice-weekly SMS messages regarding breastfeeding, with or without group messaging and access to a health professional [49]. Two studies assessed the feasibility of SMS reminders for improving immunization coverage. In Guatemala, no significant differences in coverage were found between intervention and control groups, but participants valued the reminder service, with more members of the intervention group expressing willingness to pay for it (67.5% vs 49.6%; *P* = 0.01) [50]. In Kenya, SMS reminders were combined with a cash or airtime credit incentive for timely vaccination, with results showing high coverage of pentavalent dose 1 (90%) and dose 2 (85%) [51].

Consistent with the promise shown in feasibility studies, trials of direct messaging interventions targeting care-seeking behaviors through education and reminders have primarily reported positive effects. In China, SMS reminders improved adherence to a home fortification intervention but did not produce a greater decrease in anaemia than provision of micronutrient powders without reminders (marginal effect -0.03; 95% CI = -0.09 to 0.03) [29]. Infants born to participants in a Prevention of Mother-to-Child Transmission of HIV (PMTCT) program in Kenya who also received weekly SMS messages were significantly more likely to undergo postnatal infant HIV testing than those receiving usual care (92.0% vs 85.1%; RR = 1.08; 95% CI = 1.00-1.16) [25]. The other intervention studies in this review reported effects of direct messaging interventions on uptake of maternal health services (ANC and facility-based delivery), infant immunization or exclusive breastfeeding.

In the Wired Mothers trial in Zanzibar, pregnant mothers receiving regular SMS messages were significantly more likely to attend at least four ANC visits (OR = 2.39; 95% CI = 1.03-5.55) [20]. Skilled attendance at delivery also increased significantly, but only among urban women (OR = 5.73; 95% CI = 1.51-21.81) [19]. Analysis of secondary outcomes found that perinatal mortality was significantly reduced in the intervention group (OR = 0.50; 95% CI = 0.27-0.93) [21]. In a randomized controlled trial in Kenya, mothers receiving SMS reminders and pregnancy health information were significantly less likely than the control group to have fewer than four ANC visits (3.6% vs 9.7%; *P* = 0.002) and were more likely to deliver in a health facility (88.0% vs 72.8%; *P* = 0.00), although there was no difference in intrauterine or neonatal mortality between groups [22]. In a randomized controlled trial in Ahmednagar, India, mothers receiving ANC reminder phone calls and SMS messages with pregnancy health information were significantly more likely than the control group to have at least four ANC visits (57.5% vs 23.5%; *P* < 0.0001), while facility-based delivery was high in both groups [24]. In Nigeria, a quasi-experimental study compared participants’ place of delivery with past pregnancies, and reported a greater increase in facility-based delivery in the group receiving the SMS intervention compared with the group receiving general health messages (29% vs 13%) [23]. A quasi-experimental study in rural India found that delivering IVR messages to husbands of pregnant women resulted in significantly more facility-based births in the intervention group (93.3% vs 62.1%; *P* < 0.001) but showed no effect on the proportion of mothers attending at least four ANC visits or consuming 100 iron folic acid tablets [35].

We identified three studies of SMS reminders for childhood immunization. In Bangladesh, full vaccination coverage for children over 298 days old increased in areas reached by an SMS reminder service and decreased in control communities, for a difference-in-differences of +29.5% in rural areas (*P* < 0.001) and +27.1% in the urban areas (*P* < 0.05) [26]. A cluster-randomized controlled trial in Kenya compared full vaccination rates at 12 months of age following SMS reminders, with two groups also receiving a cash incentive [28]. The group receiving SMS reminders plus the larger cash incentive (KES200; US$2.35) were significantly more likely to achieve full immunization (RR = 1.09; 95% CI = 1.02-1.16; *P* = 0.014) [28]. A quasi-experimental study comparing SMS with sticker reminders for pentavalent vaccination in rural Kenya found that children in the SMS group were significantly less likely to miss the third vaccine dose (OR = 0.2; 95% CI = 0.04-0.8) [27].

Three studies targeted breastfeeding practices, with positive effects. In China, the median duration of exclusive breastfeeding was significantly greater in the SMS-intervention group, at 11.41 weeks (95% CI = 10.25-12.57) compared with 8.87 weeks (95% CI = 7.84-9.89) for the control (*P* < 0.001) [14]. Messaging content was based on formative research findings and national guidelines, and a process evaluation found high satisfaction with the service [37]. Flax et al. also reported improvements in breastfeeding practices from a multi-dimensional BCC intervention in Nigeria, in which direct messaging to phones shared by small groups of women was integrated with large group education sessions as part of a micro-finance program [16]. The cluster randomized trial found a significantly greater likelihood of timely breastfeeding initiation (OR = 2.6; 95% CI = 1.6-4.1) and exclusive breastfeeding for six months (OR = 2.4; 95% CI = 1.4-4.0) in the intervention group [16]. Implementation research showed that the majority of participants were satisfied with group cell phones, and that mothers in groups which met at least weekly were more likely to exclusively breastfeed for six months (OR = 5.6; 95% CI = 1.6-19.7) [38]. A randomized controlled trial in Kenya comparing the effects of weekly 1-way or 2-way SMS messaging found no differences between groups for facility-based delivery or postpartum contraception use, but a significantly higher probability of exclusive breastfeeding in both intervention groups compared with the control group at ten weeks (OR = 0.93; 95% CI = 0.86-0.97 and OR = 0.96; 95% CI = 0.89-0.98 vs OR = 0.72; 95% CI = 0.69-0.86; *P* < 0.005) and 16 weeks postpartum (OR = 0.82; 95% CI = 0.72-0.89 and OR = 0.93; 95% CI = 0.85-0.97 vs OR = 0.62; 95% CI = 0.52-0.71; *P* < 0.005), with sustained higher probability of exclusive breastfeeding at 24 weeks postpartum in the 2-way SMS group (OR = 0.62; 95% CI = 0.51-0.72 vs OR = 0.41; 95% CI = 0.31-0.51; *P* = 0.005) [18].

In contrast with interventions designed for research studies, which usually focus on a very limited number of target outcomes, programmatic applications of direct messaging typically encompass multiple MNCH topics. SMS or IVR services for MNCH have been launched at a large scale through public-private partnerships in several LMIC, including Bangladesh [58], Tanzania [62], South Africa [40], India [64] and eight sub-Saharan African countries participating in the GSMA mNutrition initiative [65]. Public-private partnerships are essential to achieve sustained implementation at-scale, but challenging to build and maintain as it is not easy to align the goals and perspectives of diverse stakeholders [43,65]. These large-scale programmes deliver regular SMS or IVR messages to personal phones, with content tailored to the subscriber’s stage of pregnancy or infancy. High subscriber numbers indicate acceptability and demand for the services, but to date there is limited evidence of effectiveness for improving MNCH practices.

Analysis of system-generated data from MomConnect, a national-level maternal mHealth service in South Africa, found that half of all women attending the first ANC visit registered with the service, and 80% of messages were successfully received [40]. A rapid assessment in KwaZulu Natal province also reported high enrollment, with two-thirds of subscribers registering for MomConnect prior to 20 weeks gestation [34]. MomConnect includes both weekly SMS messaging and access to a helpdesk for subscribers to ask health questions and provide feedback on maternal health services, although implementation studies have found low awareness and usage of the helpdesk feature [41,42]. To our knowledge there is no large-scale evaluation of the effectiveness of MomConnect for changing MNCH practices, but a study of MAMA South Africa, an SMS service which preceded and informed the design of MomConnect, found that HIV+ subscribers were significantly more likely than non-subscribers to attend at least four ANC visits (RR = 1.41; 95% CI = 1.15-1.72) and less likely to deliver a low birth weight infant (RR = 0.14; 95% CI = 0.02-1.07) [31]. A subsequent case-control study with mothers in Johannesburg did not find any significant differences between MomConnect subscribers and non-subscribers, although the interpretation of findings was compromised by the very small sample size [33].

An observational study of subscribers to Aponjon, a large-scale direct messaging intervention in Bangladesh, found no differences in skilled attendance at birth, timely initiation of breastfeeding, delayed newborn bathing or postnatal care visits between those who did and did not receive Aponjon messages during pregnancy [30]. A separate retrospective observational evaluation which compared Aponjon subscribers with non-subscribers found that use of the service was significantly associated with 8 of 19 maternal practices, including ANC attendance and vitamin A supplementation post-delivery, and with neonatal cord care but not with other infant care or feeding practices [32]. Greater intensity of engagement with the Aponjon service (ie, longer length of subscription and higher reported frequency of listening to messages) was associated with greater improvements in knowledge and practices [32].

The GSMA mNutrition initiative delivers MNCH and nutrition messaging content to 1.6 million subscribers across eight countries in sub-Saharan Africa [65]. Monitoring data show that 36% of subscribers are active users of the service, with 42% reporting to share the content they receive with family and neighbours, thus increasing the reach of BCC messages [65]. A GSMA case study reported that 69% of users demonstrated correct nutrition knowledge and practices, compared with 56% and 57% of non-users, respectively, but full details of the methods for obtaining these data were not provided [65].

In contrast with the majority of direct messaging interventions which provide content to individual subscribers, JEEViKA Mobile Vaani (JVK) is an IVR platform in rural Bihar, India, which functions as a community media forum [44]. Users can listen to pre-developed content and contribute their own comments and questions, which are curated by a team of moderators. The aim of this interactive approach is to stimulate intra-household and community dialogue related to core MNCH topics in order to facilitate behavior change. Lessons learned from the first year of implementation include the need for extensive technology training and support to facilitate user adoption of the platform and the need to include non-core content of interest to users in order to increase engagement overall [44]. Implementing JVK through existing women’s self-help groups increased female participation in a setting where many women have limited access to mobile phones, and working through community volunteers increased the coverage of marginalized groups [44].

The learnings from implementation studies as well as programmatic case studies demonstrate the need for formative research to guide contextualization of direct messaging interventions, including the choice of messaging delivery mode (SMS or IVR) [58,59,64]. We identified six published formative studies for this review, but acknowledge that many other interventions were informed by unpublished formative research findings. In Myanmar, participants in a study to inform the design of a randomized trial identified specific barriers to exclusive breastfeeding, and recommended contextualized text messages sent in the evenings for the intervention [52]. Surveys in rural Kenya, Afghanistan and Peru all found high rates of mobile phone access and receptivity to direct messaging for MNCH [54-56]. However, some formative studies find that direct messaging is not a desired BCC delivery approach. Mothers living on two tea estates in Sri Lanka expressed openness to IVR, but had limited experience using mobile phones and preferred face-to-face counseling on infant and young child feeding [53]. Participants in a study in Ghana were similarly open to an IVR intervention for care-seeking but had no experience with IVR and preferred a hot-line instead [57].

Formative and implementation research are also needed to guide the development and rigorous pre-testing of localized messaging content [58-62,64]. For IVR interventions, voices used in narration are often chosen to represent locally relevant authority figures such as doctors, village leaders or grandmothers, in order to build trust and increase the persuasiveness of messaging content [58,63,64]. In Ghana, users described a process of gradually learning to trust the advice given through MOTECH, a maternal health messaging service, but needing to balance this advice with traditional practices valued in their communities [39]. Users of a direct messaging service in Tanzania identified the need to incorporate avenues for participants to connect with a health care professional for more in-depth discussion of messaging topics [61]. Some interventions have incorporated either two-way messaging [14,18] or telephone contact with a health care provider [19,42,58], but the factors contributing to effective utilization of these services in different contexts are not well understood.

### Voice counseling

There are two voice counseling approaches: i) health hotline services, in which users access the advice of a health professional through a call center; and ii) counseling initiatives which link clients with qualified counselors who pro-actively offer support, information and advice over the phone. These have not been widely tested for MNCH communication in LMIC (**Table 3**).

In rural Bangladesh, participants in a feasibility study reported improved awareness and attention to pregnancy risk management with toll-free numbers providing access to medical advice without the expense and difficulty of travel to a health facility [67]. In Quito, Ecuador, delivery of postnatal health education via mobile phone was associated with higher rates of neonatal health checks (72% vs 53.3% in the control group; *P* = 0.022) and exclusive breastfeeding at three months postpartum (86.7% vs 66.7%; *P* = 0.005) [66]. In an RCT comparing pro-active voice counseling with monthly peer support groups in Kenya, exclusive breastfeeding at three months was significantly greater in the mobile phone group (91%) compared with either the peer support group (83%) or the control (78%), which were not significantly different from each other [15]. A formative study in rural Bangladesh found that both community health workers and mothers preferred voice calls over direct messaging as a means to expand coverage of counseling on infant and young child feeding, although many mothers had limited access to mobile phones [68].

### Direct messaging + voice counseling

Fourteen papers reported on the use of direct messaging and voice counseling in combination, including five from the Chipatala Cha Pa Foni (CCPF) pilot project in Malawi (**Table 4**).

The CCPF pilot project included a toll-free hotline staffed by trained district hospital personnel and a subscriber-based ‘tips and reminders’ messaging service covering multiple MNCH topics [72]. The project aimed to improve uptake of both home-based preventive practices and facility-based health care for mothers and children under five. Over the two-year project period, more than 9000 unique users accessed the hotline and over 5800 subscribed to the messaging service, with 68% using shared phones [75]. Analysis of monitoring data showed higher message delivery rates for subscribers accessing SMS compared with IVR in this context, likely related to phone access issues [36]. Treatment-on-treated analysis of the evaluation data found a significant positive effect on the uptake of home-based care for both mothers and children, and for facility-based care for mothers but not for children [69]. The improvement in home-based care for children was driven by the increased uptake of insecticide-treated bed nets, with no changes seen in exclusive breastfeeding or use of Oral Rehydration Solution for diarrhea management [70]. A cost-outcome analysis found that CCPF costs ranged from US$67 to US$355 per pregnant user reporting improvements in MNCH knowledge or practice, and sensitivity analysis showed the potential to significantly reduce costs per outcome through implementation at full capacity [76]. Following the pilot project, CCPF has expanded in its content areas beyond MNCH and scaled up geographically, in partnership with the Ministry of Health and AirTel, with the goal of national coverage [110].

A pilot study in Iraq which combined weekly text messages with access to a hotline for pregnant mothers found a significantly higher median number of ANC visits in the intervention group (4 vs 2; *P* < 0.001), and 60.8% of mothers in the intervention group utilized the hotline service [79]. The other studies identified in this review prioritized pro-active voice counseling rather than hotline services in combination with direct messaging.

In a cluster-randomized trial of infant feeding support in India, mothers who received daily SMS messages and weekly mobile phone calls were significantly more likely to exclusively breastfeed their infants at six weeks (97% vs 80.9%; *P* < 0.001), 14 weeks (96.2% vs 70.7%; *P* < 0.001) and six months postpartum (97% vs 48.5%; *P* < 0.001), with a widening gap between the intervention and control groups at each time point [71]. A feasibility study in rural Bangladesh found high acceptance of an mHealth intervention aiming to improve infant feeding practices through IVR messaging, bi-weekly calls from an infant feeding counselor, access to a call centre and provision of a monthly unconditional cash transfer [78]. Outcome measures were not assessed, but half of the participants utilized the call centre and a majority (62.5%) indicated a preference for the combination of IVR and voice counseling as a means to receive nutrition advice [78]. A qualitative formative research study of mHealth for PMTCT services in Kenya also recommended a combined intervention approach, with direct messaging for timely delivery of neutral information and reminders but voice counseling as the preferred option for discussion of more complex or confidential information [80]. A feasibility study among adolescent mothers in Honduras identified the importance of establishing a trusting relationship through interpersonal contact prior to launching mobile phone-based breastfeeding support [81].

The Liga Inan program in Timor Leste implemented twice weekly SMS “tips and reminders” for registered pregnant mothers and facilitated two-way communication between users and their midwives, with high uptake (70% of pregnant women in the implementation area accessed the service) [77,82]. Comparison of health service utilization practices between baseline and endline surveys found large increases in facility-based delivery (32% to 49%), skilled birth attendance (48% to 62%) and timely postnatal care for both mothers and infants (26% to 51%; 20% to 39%) in the intervention area [73,74]. These indicators reportedly remained the same or deteriorated in the control area, but evaluation methods were not well described and statistical significance was not reported [73,74].

### Job aid applications

Frontline workers (FLWs) are vital to the delivery of community-level MNCH services in many LMIC [111]. Multiple projects are utilizing mobile phone-based job aid tools, primarily smartphone applications (apps), to strengthen FLWs’ performance during routine home visits to pregnant women and mothers of young children [112]. These apps typically combine efficient client registration and data tracking systems with checklists, protocols and reminders of key health topics, and prerecorded BCC messages are often embedded for sharing with clients [105,113]. The checklists and reminders are intended to increase consistency of care by prompting FLWs to discuss key topics with their clients, which can be reinforced by the embedded BCC messages. Fifteen published studies and twelve programmatic reports of mobile phone-based job aid tools were included in this scoping review (**Table 5**). Some of these reports compile evidence from multiple projects, reflecting the widespread use of job aid apps [92,105-108].

Pilot studies have found high acceptance of job aid apps by FLWs and beneficiaries, and report improvements in indicators of care delivery [95-97,104,106,109]. Preliminary findings of a randomized controlled trial in Niger are an exception; they indicate no significant improvements in assessment or counseling for priority childhood illnesses (fever, pneumonia, and diarrhea) among FLWs equipped with a job aid app for Integrated Community Case Management [92].

A single, small formative study found that greater proficiency in using a job aid app was associated with improved care delivery [98]. Evidence from qualitative studies indicates that audio or video BCC messages embedded in job aid apps increase the credibility of FLWs in the community, adding an external authoritative voice to their communication [90,100,101]. In communities with limited prior experience with technology, smartphones may be regarded with suspicion but embedded messages may help legitimize their use [100], although client concerns regarding data storage and privacy have also been reported [92,99]. Many programmatic evaluations report high acceptability and improvements in indicators of care delivery and beneficiaries’ knowledge, attitudes and practices when FLWs are equipped with job aid apps, although study methods are often inadequately reported [92,105,107,108].

One published study attempted to assess the impact of mobile phone-based job aid tools on maternal and infant mortality in rural Guatemala [83]. Methodological limitations noted for this study include a lack of data to confirm an association between reported changes in maternal mortality and the mHealth intervention [112]. However, several intervention studies have reported positive impacts related to proximal measures of maternal care. In a cluster randomized trial in Tanzania, mothers served by FLWs using a job aid app were significantly more likely to deliver in a health facility (OR = 1.96; 95% CI = 1.21-3.19; *P* = 0.01) [87]. In Zanzibar, Tanzania, facility-based delivery rates were significantly higher than Demographic and Health Survey data for the region (75% vs 35%) following implementation of a multi-function job aid app which combined client registration, tracking and counseling with a referral service and mobile money transfer to facilitate transportation to health facilities [85]. A non-randomized controlled evaluation in Ethiopia found that mothers served by health workers receiving pregnancy care information and appointment reminders through a job aid app were significantly more likely than the control group to have facility-based delivery (AOR = 1.98; 95% CI = 1.53-2.55) and attend postnatal care (AOR = 2.77; 95% CI = 2.12-3.61), but there was no significant difference in attendance at four or more ANC visits (AOR = 1.31; 95% CI = 1.00-1.72) [86]. In Nigeria, quality scores for facility-based ANC improved after implementation of a job aid app, driven by improved frequency of BCC message delivery [84]. Assessment of a job aid app in rural Uttar Pradesh, India, found significant improvements in four of eight MNCH indicators in both intervention and control groups compared with baseline regional survey data, but the intervention group had significantly greater improvements in self-reporting of illness during pregnancy (13.2 percentage points; *P* = 0.04) and after delivery (19.5 percentage points; *P* = 0.01) [89].

Two studies reported the effects of equipping FLWs with job aid apps in the context of comprehensive MNCH programming. In Jharkhand, India, mothers participating in an NGO-led MNCH program who were also counseled by FLWs using a job aid app were significantly more likely to attend at least four ANC visits (OR = 1.23; 95% CI = 1.17-1.29) and to deliver in a health facility (OR = 1.19; 95% CI = 1.13-1.25) compared with those receiving the MNCH services but not the mHealth component [88]. A process evaluation of this project found that although the apps increased FLWs’ confidence and efficiency, the impact on uptake of maternal care seeking was limited by barriers related to household resources, power dynamics and women’s workload [94]. In the Ananya program in Bihar, India, significant differences in several health practices were reported, with the greatest effect for three or more ANC visits among mothers served by FLWs using a job aid app (50% vs 29%; *P* < 0.001) [90]. Comparison of program monitoring data with government statistics for the remaining districts of Bihar found higher coverage of MNCH services in implementation areas where FLWs were equipped with the smartphone job aid app, but statistical significance was not assessed [93].

Mobile Kunji, a second job aid tool initially designed and deployed in the Ananya program, leverages the basic phones already owned by FLWs, rather than introducing smartphone technology [102]. BCC messages are communicated first through a set of visual aids carried by the FLW. Each card in the visual aid tool has a unique code to access an IVR message via mobile phone, reinforcing the message on the card. Guided by formative research findings, IVR messages are delivered in the voice of a female doctor (“Dr. Anita”) in order to create an authoritative yet warm connection with listeners [102]. An evaluation found a greater likelihood of mothers saving the FLW’s phone number (OR = 2.72) and feeding their 6-11 month-old child at least one food group in previous 24 hours (OR = 1.72) if they were exposed to the Mobile Kunji messages [91].

Both formative studies and intervention evaluations of job aid apps highlight the need for extensive and on-going technical training and support to FLWs when novel technology is introduced [90,97,106,108]. Full mastery of app functions may occur in stages and takes time, which may not be compatible with relatively short project timeframes. There is also a need to build competencies in interpersonal counseling so that BCC tools are used to their full potential [90,97,103,105,107].

### Interactive media

We included a fourth category to acknowledge the emergence of more advanced technology in LMIC, including smartphones and feature phones with internet capabilities. We chose the term interactive media to reflect the distinctive features of the newer technology, which allows users to interact multilaterally with one another, and to generate and distribute content.

We found only one intervention study of an interactive media intervention for MNCH in LMIC (**Table 6**). A pilot test of a smartphone app for improving Indonesian husbands’ birth preparedness/complication readiness (BP/CR) scores found significantly higher scores in the intervention group both three weeks after intervention delivery (72.9 vs 62.6; *P* = 0.001) and in the postpartum test (81.8 vs 71.3; *P* < 0.001) [114]. Although this was the only published study identified, there are several project examples. These include the Alive & Thrive program in Vietnam, whose website offering online infant and young child feeding counseling and an interactive forum received more than 1 million unique visitors over 26 months [115]. In India, HealthPhone^TM^ has launched free nutrition apps in multiple Indian languages as well as providing an extensive health and nutrition video library that is available via microSD card or can be preloaded into low-cost mobile phones [116].

## DISCUSSION

### Building the mHealth evidence base

As the mHealth field matures, there is a growing need as well as increased ability to categorize and examine interventions according to their specific approaches and targeted health outcomes, rather than trying to determine a collective effect [3]. This more granular analysis will provide much needed clarity on what works where for mHealth. Effectiveness evidence must also be complemented with implementation science research to complete the picture of *how* mHealth interventions work in various contexts [117]. This scoping review therefore explored the evidence for the use of mobile phones for MNCH BCC delivery from both effectiveness and implementation perspectives.

The evidence base on the effectiveness of BCC delivery approaches using mobile phones is growing but remains limited for many MNCH outcomes. The most widely studied outcomes relate to improved uptake of maternal health services, specifically ANC attendance and facility-based delivery. Other reviews have found sufficient evidence of effectiveness of mHealth BCC delivery for these outcomes [5,12,13]. While there is room for continued investigation on these topics, an increased focus on building the evidence base for other MNCH outcomes and for the effectiveness of interventions addressing multiple MNCH practices is warranted.

There is also a need for more examination of voice counseling and interactive media as BCC delivery approaches for MNCH. The majority of the effectiveness studies we identified utilized either direct messaging or job aids as the BCC delivery approach. This is anticipated because the use of mHealth approaches for BCC began with direct messaging interventions delivered to the most basic mobile phones, and SMS continues to be widely used in LMIC. With the advent of smartphone technology and open source software platforms, many agencies and projects have introduced job aid apps for FLWs [112]. We identified only two randomized trials for voice counseling, one for direct messaging combined with voice counseling and none for interactive media. There remains an acute need for new research to further develop the evidence base, including trials comparing and combining delivery approaches.

### Implementation elements

All BCC intervention designs must be contextualized, so that both the delivery channel and the content are appropriate for the target audience, as emphasized in several case studies and project reports included in this review [58,61,102,103]. We identified four delivery channels through which mobile phones are used for BCC: direct messaging, voice counseling, job aid applications and interactive media. These four channels differ markedly from one another in several core implementation elements which affect the intensity of BCC delivery and are therefore likely to mediate its effect on MNCH practices. These include the frequency of communication, length and complexity of content delivered, and potential for personalization.

Direct messaging provides brief, standardized content distributed on a regular schedule, often weekly. Messages can be tailored to the recipient’s stage of pregnancy or infancy but additional personalization is limited. Trials of direct messaging interventions with a primary focus on one MNCH practice have shown evidence of effectiveness in improving uptake of maternal care services [19,20,22,24], childhood immunization [26,28] and exclusive breastfeeding [14,16,18]. Direct messaging is attractive because of its potential for high coverage, and several large-scale direct messaging programmes have been successfully launched [58,62,64,65]. However, it is unclear whether the positive effects reported in experimental studies focused on very specific MNCH practices will be realized in programmatic implementation of more comprehensive MNCH messaging. Preliminary findings from the Aponjon evaluation in Bangladesh suggest improvements in some maternal care practices among users of the service, but little effect on infant care or feeding practices [32], while analysis of system monitoring data did not find significant differences in practices between users based on exposure to prenatal messages [30]. Further evaluations are needed to bring clarity to the question of which MNCH practices are most amenable to change through large-scale direct messaging services.

It is likely that brief, standardized messaging services will be more effective for episodic behaviours (such as attendance for ANC or immunization) while habitual practices with complex, socially-mediated determinants (such as exclusive breastfeeding) will require more intensive and multi-faceted BCC interventions [1]. An analysis of behaviour change techniques for improved child health in LMIC found that successful projects utilized a variety of techniques in addition to information provision in order to engage, motivate and enable participants to adopt and sustain new behaviours [2]. These considerations are relevant to the selection of mHealth approaches for BCC intervention designs. We found voice counseling to be the least utilized BCC delivery approach, yet it offers a vehicle for interpersonal support delivered on a flexible timing basis, thereby increasing both the intensity and personalization of content.

Combining direct messaging with voice counseling allows for consistent reinforcement of core messages complemented by personalized support. This approach was effective for improving breastfeeding practices in India [71], while in the CCPF project in Malawi, the combination of direct messaging with responsive voice counseling through a health hotline showed mixed results, with significant improvements in bed net use but not in exclusive breastfeeding [70]. Further research is needed to compare the relative effectiveness and cost-effectiveness of different combinations of direct messaging and voice counseling delivery approaches for specific MNCH outcomes in various contexts.

Job aid apps operate on a continuum of intensity, depending on the counseling capacity of FLWs. At a minimum, the BCC messages embedded within most apps provide brief, standardized content similar to direct messaging. For the reasons outlined above, these messages alone are unlikely to influence meaningful behaviour change, but several projects in this review contribute evidence that the messages in job aid apps can reinforce and enhance the effectiveness of counseling provided by FLWs with adequate skills, time and support to facilitate more in-depth, personalized discussion of the basic messages for enhanced BCC delivery [91,101,104]. It is therefore important to combine training and support for the deployment of job aid apps with ongoing investments in FLW counseling capacity [102,103].

Only one study of an interactive media intervention was identified for this review, although use of this delivery channel is likely to expand dramatically as smartphone and internet access continue to increase in LMIC [6]. Interactive media offers the opportunity for rich, multi-directional engagement with health content, but often without any involvement of health professionals. New models of engagement, quality assurance and evaluation are required to leverage the new opportunities interactive media creates, and to mitigate potential harm from erroneous content [118,119].

We suggest there are many reasons to consider these differences in implementation elements when designing interventions and interpreting effectiveness data. In particular, expectations for changes in complex behaviors must be realistic and linked to the intensity of BCC delivery and the degree of personalized problem-solving support that is provided [1,2]. Embedding mHealth BCC within multi-dimensional interventions may help increase engagement and overcome limitations of specific mHealth delivery channels as well as addressing multiple drivers of target MNCH practices [16,44,85]. The differences between the mHealth delivery approaches also have implications for allocation of human, financial and technical resources. Job aid apps are deployed through the existing workforce of FLWs, while implementing voice counseling services may require recruitment and training of new personnel. Quality assurance is managed through the content development process for direct messaging and interactive media, and through investment in building the capacity of frontline service providers for voice counseling and job aid apps. Direct messaging and voice counseling utilize the phones already owned by users. Negotiating subsidized airtime rates or toll-free calling facilitates inclusion of users in the poorest quintiles [64]. Interactive media interventions leverage users’ access to smartphone and internet technology. In contrast, interventions deploying job aid apps typically provide smartphones to FLWs. This requires a concurrent investment in training and support for adoption of new technology, and has implications for sustainability [102,113].

### Limitations

We conducted extensive searching for this scoping review, but some eligible interventions may have been missed, particularly from the programming domain. Searching the mHealth evidence base is complicated by the wide dispersal of relevant papers across both the published and grey literature. We aimed to mitigate this challenge by using broad search terms and including multiple online repositories of mHealth documents in the grey literature search, as well as leveraging networks of mHealth implementers to identify relevant projects. However, many mHealth programs are unevaluated or unreported and therefore unavailable for review, and we were not able to include documents in languages other than English. There are many additional examples of projects implementing BCC for MNCH using mobile phones but those without documented evaluation data or reflections on lessons learned were excluded.

This scoping review focused on BCC interventions for MNCH delivered via mobile phone to mothers and families during pregnancy and the first five years of childhood. mHealth interventions targeting other stages of the lifecycle or designed to build FLW capacity, improve adherence to protocols or strengthen communication between health providers were therefore not included, but may contribute to improved quality of MNCH BCC delivery.

The shortage of rigorously evaluated mHealth interventions is a persistent challenge noted by many reviewers [5,12,13]. This scoping review was similarly constrained by the relatively small number of eligible studies related to voice counseling and interactive media, and the wide variation between designs and intervention contexts overall. However, the aim of a scoping review is to map the existing state of evidence in order to provide direction for future development of the field, and this was achieved.

### Recommendations for interventions

The recently released Digital Health Guidelines provide comprehensive guidance for mHealth interventions [5]. The program examples we reviewed align with global guidance and emphasize in particular the following principles for BCC intervention designs utilizing mobile phones:

- Align both the content and the delivery channel to the context, based on formative research.
- Ensure BCC messages are developed and tested with local partners so they will be relevant, understandable and appealing to the target audience.
- Use technology that is already familiar to the target users. If new technology will be introduced, be prepared to provide ongoing training and support throughout implementation.
- Allow sufficient time for behavior change, especially on complex, habitual behaviors. The relatively short duration of many mHealth projects does not allow a long enough implementation phase after the design is tested and deployed.

### Recommendations for research

The evidence base for mHealth approaches for BCC delivery is growing but critical gaps remain. We propose the following five areas for further research:

- Effectiveness studies (trials or rigorous programmatic evaluations) of specific mHealth delivery channels targeting specific MNCH outcomes, particularly studies comparing voice counseling and direct messaging implemented both singly and in combination.
- Cost-effectiveness studies embedded within effectiveness studies, including comparisons of voice counseling and direct messaging implemented both singly and in combination, and comparison of hotline services with pro-active voice counseling.
- Theory-based Program Impact Pathway analyses to enhance understanding of how mHealth delivery approaches contribute to improved MNCH practices and outcomes.
- Implementation Science research, including project process evaluations, to inform best practice guidance with a greater understanding of barriers and enablers to the effective use of mHealth approaches.
- Evaluation and Quality Assurance methods and tools need to be developed and tested for use with interactive media interventions.

## CONCLUSION

There is a need for granular analysis of specific mHealth intervention approaches in order to inform best practice guidance. We identified four different delivery approaches for MNCH BCC using mobile phones, and reviewed the evidence for each from both effectiveness and implementation perspectives. The effectiveness evidence base remains limited but is growing, particularly for direct messaging and job aid interventions, and we propose five areas for further research. The four mHealth approaches differ in key implementation elements, with implications for intensity of BCC delivery and other program design considerations. BCC should always be contextualized both in terms of content and delivery approaches, guided by formative research.

## Additional material

Online Supplementary Document
