# Supplementary material for: Use of mobile phones for behavior change communication to improve maternal, newborn and child health: a scoping review
Source: J Glob Health. 2019 Dec 16;9(2):020425. doi: 10.7189/jogh.09.020425 (PMC6925966; doi:10.7189/jogh.09.020425)
Supplement: Online Supplementary Document [file jogh-09-020425-s001.pdf]

Appendix S1.

PubMed Full Search Strategy

((((("counselling"[All Fields] OR "counseling"[MeSH Terms] OR "counseling"[All Fields]) OR (("behavior"[All Fields] OR "behavior"[MeSH Terms] OR "behavior"[All Fields]) AND ("Change"[Journal] OR "change"[All Fields]))) OR ("nutritional status"[MeSH Terms] OR ("nutritional"[All Fields] AND "status"[All Fields]) OR "nutritional status"[All Fields] OR "nutrition"[All Fields] OR "nutritional sciences"[MeSH Terms] OR ("nutritional"[All Fields] AND "sciences"[All Fields]) OR "nutritional sciences"[All Fields])) OR (("infant"[MeSH Terms] OR "infant"[All Fields]) AND feeding[All Fields])) OR ("breast feeding"[MeSH Terms] OR ("breast"[All Fields] AND "feeding"[All Fields]) OR "breast feeding"[All Fields] OR "breastfeeding"[All Fields])) AND ("cell phones"[MeSH Terms] OR ("cell"[All Fields] AND "phones"[All Fields]) OR "cell phones"[All Fields] OR ("mobile"[All Fields] AND "phone"[All Fields]) OR "mobile phone"[All Fields]) AND English[lang]

Table S1. Grey Literature Sources: mHealth Online Repositories and Databases

Center for Health Market Innovations
GSMA Mobile for Development Resources
Health Enabled
Johns Hopkins University Global mHealth Initiative
Kiwanji
mHealthevidence
mHealth Knowledge
mHealth Working Group project inventory
NetHope Solutions Center
USAID mHealth Compendiums
WHO eHealth database

Table S2. Grey Literature Sources: Websites of Organizations or Initiatives

Alive & Thrive
Ananya<br>Shaping Demand and Practices
BBC Media Action
CORE Group<br>CORE Group mHealth Interest Group
Dimagi
GramVaani
Grand Challenges
GSMA mNutrition Initiative
Health Alliance International
Health Information for All
Human Network International
HealthPhone
ImTeCHO
Institute of Development Studies
Jacaranda Health
John Snow, Inc.
Medic Mobile
Mira Channel
Mobenzi Researcher
Mobile Alliance for Maternal Action
mPowering Frontline Workers
mSAKHI
Nurture Project International
South Asia Infant Feeding Research Network
Tula Salud
World Vision International
